# *Rhodnius*, Golden Oil, and *Met*: A History of Juvenile Hormone Research

**DOI:** 10.3389/fcell.2020.00679

**Published:** 2020-08-07

**Authors:** Lynn M. Riddiford

**Affiliations:** Department of Biology, Friday Harbor Laboratories, University of Washington, Friday Harbor, WA, United States

**Keywords:** juvenile hormone, *Rhodnius prolixus*, *Hyalophora cecropia*, *Manduca sexta*, *Methoprene-tolerant*

## Abstract

Juvenile hormone (JH) is a unique sesquiterpenoid hormone which regulates both insect metamorphosis and insect reproduction. It also may be utilized by some insects to mediate polyphenisms and other life history events that are environmentally regulated. This article details the history of the research on this versatile hormone that began with studies by V. B. Wigglesworth on the “kissing bug” *Rhodnius prolixus* in 1934, through the discovery of a natural source of JH in the abdomen of male *Hyalophora cecropia* moths by C. M. Williams that allowed its isolation (“golden oil”) and identification, to the recent research on its receptor, termed *Methoprene-tolerant* (*Met*). Our present knowledge of cellular actions of JH in metamorphosis springs primarily from studies on *Rhodnius* and the tobacco hornworm *Manduca sexta*, with recent studies on the flour beetle *Tribolium castaneum*, the silkworm *Bombyx mori*, and the fruit fly *Drosophila melanogaster* contributing to the molecular understanding of these actions. Many questions still need to be resolved including the molecular basis of competence to metamorphose, differential tissue responses to JH, and the interaction of nutrition and other environmental signals regulating JH synthesis and degradation.

## Introduction

Serendipity is often the key to novel and fundamental discoveries. Physiologists early on are taught the August Krogh Principle: “For a large number of problems there will be some animal of choice or a few such animals on which it can be most conveniently studied” (Krogh, 1929). The history of juvenile hormone (JH) research is filled with examples of this as will be clear in the following account.

Experimental endocrinology began with Berthold who in 1849 transplanted testes into castrated roosters and showed that they caused the return of the normal secondary sex characteristics (enlarged combs and wattles) (Soma, 2006). In 1856, Brown-Séquard showed that the adrenal gland was essential to life (Aminoff, 2017), then later showed that mammalian testicular extracts influence secondary sex characteristics in other animals and purportedly aging in men as well (Brown-Séquard, 1889). The first work on hormonal control of metamorphosis was that of Gudernatsch (1912) who found that extracts of mammalian thyroid were sufficient to cause precocious metamorphosis of frog tadpoles, indicating a universality of function of these extracts. All these findings showed that endocrine organ extracts contained substances that when injected into the blood could act on particular target organ(s); these substances were named “hormones” (Starling, 1905).

Stefan Kopeć working in Cracow, Poland between 1908 and 1912 was the first to show that in insects, unlike in birds and mammals, the secondary sex characteristics were not dependent on gonadal hormones (Cymborowski, 1981). He went on to demonstrate in the gypsy moth, *Lymantria dispar*, that the brain secreted a hormone that was necessary for metamorphosis (Kopeć, 1917, 1922). This “brain hormone” was the first known neurosecretory hormone in insects and was later called “prothoracicotropic hormone” (PTTH) when the particular pair of cells was isolated and shown to stimulate ecdysone secretion by the prothoracic glands of the tobacco hornworm, *Manduca sexta*, *in vitro* (Agui et al., 1979).

## Wigglesworth and *Rhodnius*

**FIGURE 1:**
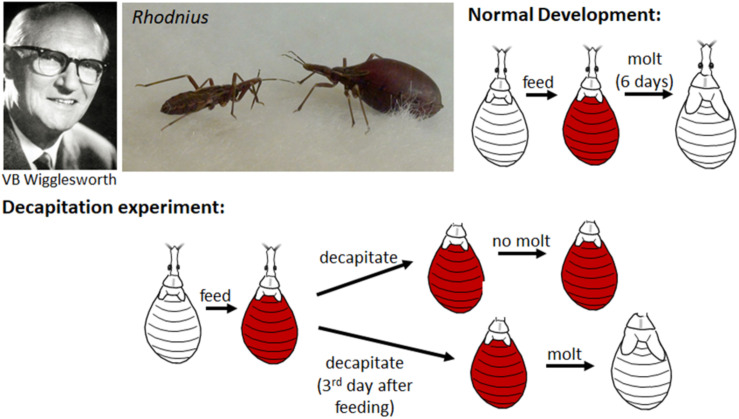
**Top**: Vincent B. Wigglesworth (left); non-fed (left) and blood-fed (right) nymphs of *Rhodnius prolixus* (center); cartoon of the normal development of a nymph which after feeding on a blood meal (orange) molts to the next nymphal instar 6 days later. **Bottom**: Cartoon of decapitation experiments shows that decapitation immediately after feeding prevents the molting but decapitation 3 days after feeding does not. Photos of Wigglesworth from Phillips (2004) and of *Rhodnius* taken by Timothy Bradley and Catherine Loudon from Knight (2014) (reproduced with permission).

**FIGURE 2:**
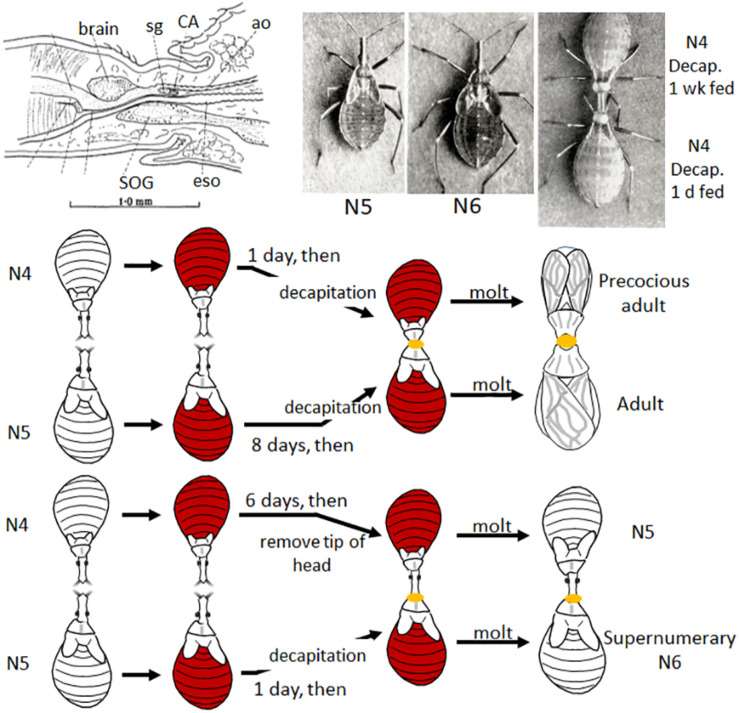
**Top**: Diagram of brain and corpus allatum (CA) of *Rhodnius* (modified from Figure 2 in Wigglesworth, 1934); *Rhodnius* 5th instar (N5), supernumerary 6th instar (N6), and parabiosed, fed and decapitated (decap) 4th instar (N4) (top one decapitated one week after feeding, the bottom 1 day after feeding) nymphs [photos from Plates I and II in Wigglesworth (1954), reproduced with permission]. **Bottom**: Cartoon of two parabiosis experiments: (1) (top) a 4th instar nymph decapitated 1 day after feeding and parabiosed to a 5th instar nymph decapitated 8 days after feeding (thus ready to molt) molted to a precocious adult; (2) a parabiosed 4th instar nymph with only the tip of the head removed (leaving the CA intact) 6 days after feeding parabiosed to a 5th instar nymph decapitated only 1 day after feeding caused the latter to molt to a supernumerary 6th instar nymph (Wigglesworth, 1934). See text for details. ao, aorta; eso, esophagus; sg, sympathetic ganglion; SOG, subesophageal ganglion.

In 1934, working with the bloodsucking, kissing bug, *Rhodnius prolixus* (the vector of Chagas disease), Vincent B. Wigglesworth was the first to show that there was a hormone that circulated in the nymphal hemolymph that prevented metamorphosis. *Rhodnius* was ideal for these experiments since they only molted after a blood meal and they could be parabiosed (i.e., two could be attached together by the anterior ends so that they shared circulating hemolymph). Normally a nymph would molt 6 days after feeding, but when decapitated immediately after feeding, it never molted (Wigglesworth, 1934; Figure 1). If, however, decapitation was delayed until 3 days after feeding, the molt proceeded normally. Through a series of parabiosis experiments, Wigglesworth (1934) then showed that there was a hormone released from the head after feeding that initiated the molting process. Moreover, parabiosis experiments with penultimate fourth and final fifth instar nymphs showed that there was also a hormone released from the head region that inhibited metamorphosis (Figure 2). Parabiosis of a fed headless fourth instar to a headless metamorphosing nymph caused precocious metamorphosis of the fourth instar nymph. In contrast, a fed headless fifth instar nymph parabiosed to a molting fourth instar nymph formed a supernumerary nymph. Histological studies showed that the head contained a brain and the posterior sympathetic ganglion that innervated a single median gland, the corpus allatum, located at the back of the head above the subesophageal ganglion in a hemolymph-filled sinus (Figure 2). Moreover, the corpus allatum showed cyclical changes during larval molting and metamorphosis. Based on these studies, Wigglesworth (1934) concluded that both the “molting” and “inhibitory” hormones come from the corpus allatum.

From further parabiosis and corpus allatum implantation experiments, Wigglesworth (1936) concluded that the corpus allatum was the source of the inhibitory hormone for metamorphosis. In 1940, he further showed by implantations of various organs into fed decapitated fourth instar nymphs that only the corpus allatum secreted the “inhibitory hormone” and the brain (the dorsal half) the “molting hormone” (Wigglesworth, 1940). At this point, he named the metamorphosis-inhibitory hormone “juvenile hormone.” He also compared the abdominal epidermal changes during a nymphal molt and at metamorphosis. Based on this comparison, Wigglesworth hypothesized that metamorphosis at the cellular level occurs only when the molting hormone is present and activates the “imaginal system” (Wigglesworth, 1940). If, however, JH is also present, it activates the nymphal system and prevents the production of adult structures. Later guided by the findings of Williams (1947, 1948) on the wild silkmoth, *Hyalophora* (formerly *Platysamia*) *cecropia* (commonly called Cecropia) (see below), Wigglesworth (1952) showed that the thoracic glands of *Rhodnius* secreted the molting hormone after activation by a hormone from the brain.

Wigglesworth also showed that the corpus allatum reactivated in the adult and regulated ovarian maturation in the female and accessory gland development in the male (Wigglesworth, 1936). The corpora allata were first described in the goat moth caterpillar, *Cossus cossus*, as “petits ganglions de la tete” (Lyonet, 1762). Later Nabert (1912) described their structure for many different insects, but their function(s) was unknown. Holmgren in 1909 and Ito in 1918 had noted changes in size of the corpora allata associated with reproductive maturation in termites and Lepidoptera, respectively (as cited in Wigglesworth, 1985), but no one had experimentally tackled the problem. Thus, Wigglesworth was the first to demonstrate that a hormone regulated reproduction in insects; and, moreover, that hormone was JH, the same hormone that regulated metamorphosis.

His findings spawned a whole series of experiments in many different insects on the role of the corpora allata in reproduction. In most insects, the corpora allata were found necessary for ovarian maturation —the grasshopper, *Melanoplus differentialis* (Pfeiffer, 1939), various flies (Thomsen, 1940; Vogt, 1942), and the cockroach, *Leucophaea maderae* (Scharrer, 1946). However, in a few insects such as the walking stick, *Carausius* (formerly *Dixippus*) *morosus* (Pflugfelder, 1937) and the silkmoth, *Bombyx mori* (Bounhiol, 1939), egg development occurred normally after allatectomy. These early studies were also notable for the number of women working in this area–Isabella Pfeiffer, Ellen Thomsen, Marguerite Vogt, and Berta Scharrer. Studies since then show that JH plays nearly a universal role in the regulation of insect reproduction although the details of the precise role it plays depends on the insect’s life history (see reviews by Wigglesworth, 1985; Wyatt and Davey, 1996; Raikhel et al., 2005; Santos et al., 2019).

## Confirmation of the Corpora Allata as the Source of JH

**FIGURE 3:**
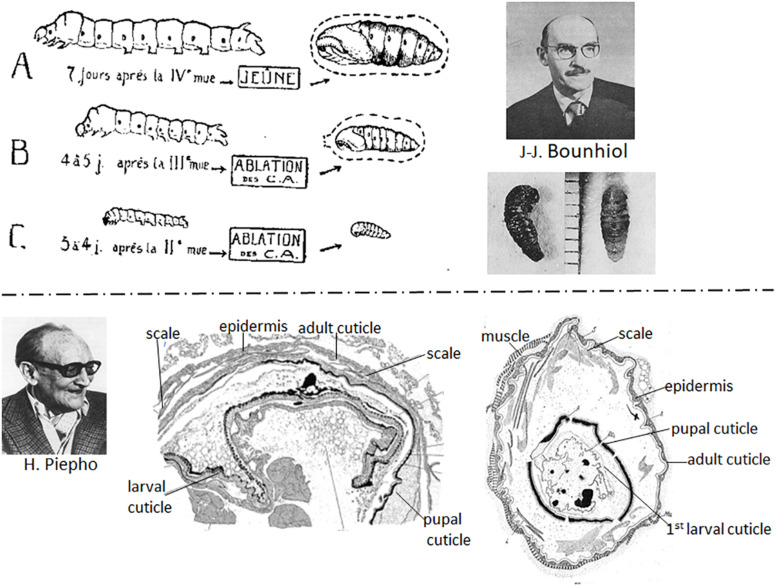
**Top**: (Left) Diagram of effects of removal of the corpora allata (CA) (allatectomy) from the silkworm *Bombyx mori*. (A) Normal 5th instar larva and pupa; (B) allatectomized 4th instar larva formed a precocious pupa; (C) allatectomized 3rd instar larva formed a precocious pupa (figure from Bounhiol, 1938). (Right) Jean-Jacques Bounhiol (above); photo of pupae formed from allatectomized 4th (left) and 3rd (right) instar larvae (from Bounhiol, 1938) (bottom). **Bottom**: (Left) Hans Piepho. (Center) Result of an implant of 5th instar *Galleria* integument (epidermis plus cuticle) into another 5th instar *Galleria* larva that subsequently metamorphosed to a pupa and an adult. The implanted epidermis formed a pupal cuticle, then an adult cuticle with scales (from Piepho, 1938a). (Right) Result of an implant of 1^st^ instar integument into a 5th instar larva after metamorphosis of the host. The implant formed a pupal, then an adult cuticle (from Piepho, 1938b). Photo of Bounhiol from Lamy and Delsol (1980) (reproduced with permission from Elsevier Masson) and that of Piepho from Hintze-Podufal (1996) (reproduced with permission from www.schweizerbart.de/journals/entomologia).

Bounhiol (1938) working with the silkworm, *B. mori*, showed that removal of the corpora allata (allatectomy) from early instar larvae caused precocious metamorphosis but removal in the final (fifth) instar had no effect on the onset of metamorphosis (Figure 3, top). The allatectomized larva formed a normal cocoon and subsequently a normal pupa and adult. At about the same time, Pflugfelder (1937) and Radtke (1942) showed that loss of the corpora allata caused precocious metamorphosis in *Carausius*, and the mealworm, *Tenebrio molitor*, respectively.

At the end of the 1930s, Piepho (1938a) and Kühn and Piepho (1940) used larval integumental (epidermis plus overlying cuticle) implants in *Galleria* larvae to study hormonal control of molting and metamorphosis. The implants molted with the host and produced the type of cuticle dictated by the hormonal environment. Thus, implants from both last instar larvae (Figure 3, bottom center) and first instar larvae (Figure 3, bottom right) that were placed in a final instar larval host metamorphosed with the host.

Also, in the late 1930s, Helen Tsui-ying Lee at Sun Yat-sen University in Canton, China studied the prothoracic glands of lepidopteran larvae and their innervation and was the first to suggest that these glands were endocrine in nature and worthy of study by insect physiologists (Lee, 1948). However, her study was not published until 1948 due to the Japanese invasion of China in 1938. By the time of the publication of her article, the Japanese scientist Soichi Fukuda had published his classic articles on the commercial silkworm, *B. mori*, showing the role of the prothoracic glands in pupation (Fukuda, 1940a, b) and of both the corpora allata and prothoracic glands in larval molting (Fukuda, 1944).

## Williams and *Hyalophora cecropia* (Cecropia), a Natural Source of JH

Carroll M. Williams grew up in Richmond, VA and loved to play around the James River that ran through the city. There he encountered among the insects he collected, the large saturniid moth, *H. cecropia*, whose pupal quiescence (diapause) inside its cocoon over the winter intrigued him. When he began his postdoctoral studies as a Junior Fellow in the Department of Biology at Harvard University in 1941, he started to work on this problem. His first studies concerned the role of the brain and prothoracic glands [recently implicated by Wigglesworth (1940) and Bounhiol (1945) to be involved in the metamorphosis of *Rhodnius* and *Bombyx*, respectively] in promoting development of the Cecropia pupa into the moth in the spring. In a series of extirpation and implantation experiments, he showed that the brain was responsible for activating the prothoracic glands to cause adult development (Williams, 1946, 1947, 1948, 1952). Moreover, chilling the pupae at 6°C for at least 10 weeks was sufficient to allow activation of the brain when the pupae were brought back to room temperature (Williams, 1956a).

### Serendipitous Discovery of a Natural Source of JH

**FIGURE 4:**
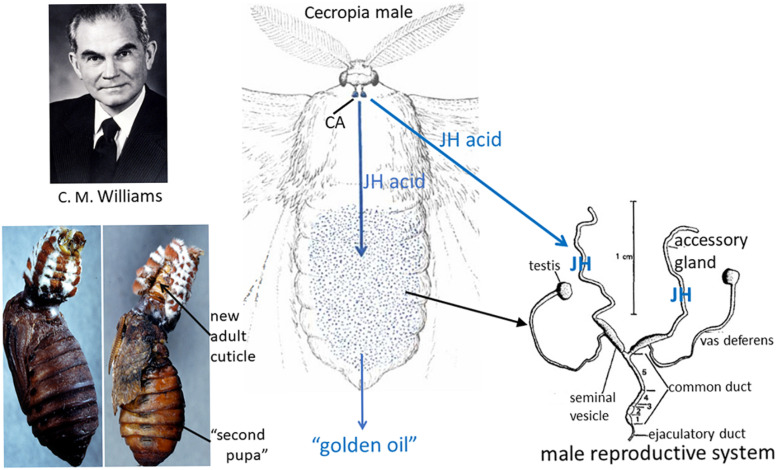
**Left**: (Top) Carroll M. Williams. Photo from his memoir in the National Academy of Sciences Memoir collection. (Bottom) Cecropia male abdomen parabiosed to a chilled diapausing Cecropia pupa (left); after 21 days, the “second pupa” had formed and the abdomen had molted to a scaleless adult abdomen (right, arrow) (from the original slides taken by Muriel V. Williams that were used for the black-and-white Figures 3, 4 in Williams, 1963). **Center**: Diagram of the Cecropia male moth whose corpora allata (CA) synthesize and secrete juvenile hormone (JH) acid and from whose abdomen the “golden oil” that contained JH was extracted (drawing of the moth modified from a figure in Williams, 1958). **Right**: Reproductive system of a male saturniid moth (modified Figure 1 in Shepherd, 1974). The accessory glands of Cecropia males synthesize JH from the JH acid that is secreted from the CA, then store the hormone (Shirk et al., 1976; Peter et al., 1981).

In the experiments described above on the role of the brain in terminating diapause, Williams became intrigued with the technique of parabiosis that Wigglesworth had used. Saturniid moths do not feed as adults so mate, lay their eggs and die within about 10 days. Therefore, Williams hypothesized that parabiosis of an adult to a pupa might allow the moth to live longer. When he parabiosed a headless Cecropia moth to a chilled diapausing pupa and kept the pair at 25°C, the moth did live longer. To his surprise, however, the pupa developed into a “second pupa” rather than to a normal adult! Although this experiment were done in the early 1950s, it was not published until 1963 (Williams, 1963). In a series of experiments to explore this phenomenon, he discovered that the “second pupa” was only formed when the parabiotic partner was either a Cecropia or a *Samia cynthia* (Cynthia) male moth (Williams, 1959, 1963; Figure 4). Cecropia pupae parabiosed to female moths of either species or to *Antheraea polyphemus* (Polyphemus) moths developed into normal adults. Moreover, the abdomens of the male Cecropia or Cynthia moths were sufficient to cause the formation of the second pupae and therefore were the repository for the “juvenile hormone” from the corpora allata that was responsible for the phenomenon (Williams, 1956b, 1959, 1963).

**FIGURE 5:**
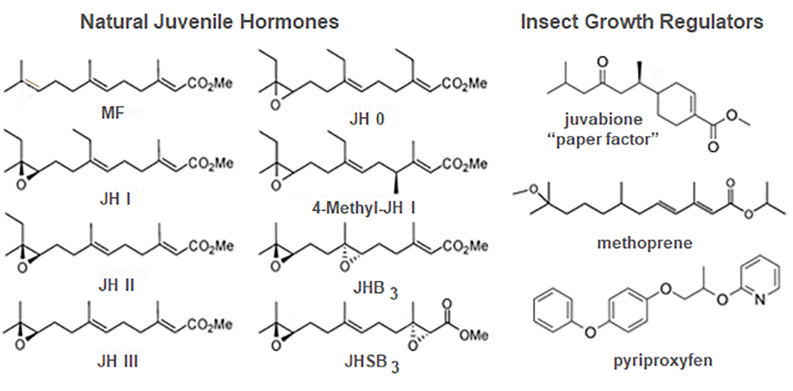
Structures of the natural juvenile hormones (JH) **(left)** and some JH analogs known as insect growth regulators (IGRs) **(right)**. JHB_3_, JH bisepoxide; JHSB_3_, JH skipped bisepoxide; MF, methyl farnesoate. Prepared by Xavier Belles.

He found that chilled Polyphemus pupae were the best assay animals for JH activity (Williams, 1959) and proceeded to make ether extracts of Cecropia abdomens to isolate the hormone (Williams, 1956b; Figure 4). This extract was yellow due to the carotenoids in Cecropia fat body so he called it the “golden oil.” During his sabbatical year in Wigglesworth’s laboratory in Cambridge, he further purified and characterized the “golden oil” (Williams, 1956b). Using this extract, Wigglesworth painted his initials VBW on abraded cuticle of final instar nymphs of *Rhodnius* and showed that these initials were present as newly synthesized nymphal cuticle surrounded by adult cuticle after metamorphosis (Wigglesworth, 1958), confirming that this hormone could act on various insect orders. The JH in this “golden oil” was not chemically determined until 1967 when Röller et al. (1967) at the University of Wisconsin identified the active compound as the sesquiterpenoid methyl *dl-trans,trans,cis*-10-epoxy-7-ethyl-3,11-dimethyl-2,6-tridecadienoate (JH I) (for a review of the chemistry, see Röller and Dahm, 1968; Figure 5) and subsequently showed that the same compound was released from male Cecropia corpora allata *in vitro* (Röller and Dahm, 1970). Meyer et al. (1968) found a second hormone, JH II (methyl *dl-trans,trans,cis*-10,11-epoxy-3, 7, 11-trimethyl-2,6-tridecadienoate) (Figure 5) as a minor component of the “golden oil.” Nearly 10 years later Shirk et al. (1976) showed that the JH was stored in the male accessory gland, not in the abdominal fat body (Figure 4). Moreover, the male corpora allata secreted JH acid that was then converted into JH by JH esterase in the accessory gland (Peter et al., 1981).

Juvenile hormones I and II were subsequently shown to be found only in Lepidoptera (Schooley et al., 1984). JH III (Figure 5) was first isolated by culturing adult female corpora allata of another lepidopteran, the tobacco hawk moth, *M. sexta* (Judy et al., 1973) and subsequently shown to be the JH of most other insects (Schooley et al., 1984). JH 0 and 4-methyl-JH I (Figure 5) were found in *Manduca* embryos (Bergot et al., 1980). JHs with modifications of the epoxide moiety are found in the Diptera and Hemiptera. JH III bisepoxide (JHB3) was first identified in *Drosophila melanogaster* (Richard et al., 1989), and JH III skipped bisepoxide (JHSB3) was first found in the stink bug, *Plautia stali* (Kotaki et al., 2009; Figure 5).

### Insect Growth Regulators

In his first article on the extraction of JH from the Cecropia male abdomen, Williams also commented on the efficacy of topical application of this extract in disrupting metamorphosis. He then suggested that “…in addition to the theoretical interest of the JH, it seems likely that the hormone, when identified and synthesized, will prove to be an effective insecticide. This prospect is worthy of attention because insects can scarcely evolve a resistance to their own hormone” (Williams, 1956b). In 1964, Karel Sláma from Czechoslovakia came to the Williams laboratory at Harvard University to work bringing his favorite study insect, the linden bug, *Pyrrhocoris apterus*. Although he brought linden seeds to feed the bugs, he used glass jars containing an upright paper towel to mimic a tree to rear the insects. Surprisingly, none of the nymphs metamorphosed under these conditions. Initially suspecting that JH might be airborne within the Williams laboratory, Sláma reared at home some *Pyrrhocoris* shipped directly from Czechoslovakia but again no metamorphosis occurred; only supernumerary nymphs appeared. They then started a systematic search for a source of the hormone in the rearing conditions that resulted in the discovery that the source was the paper towels in the rearing jars (Sláma and Williams, 1965, 1966; Williams and Sláma, 1966). It proved only to be in American, not European, paper towels because the American paper towels and other paper products were made from balsam fir and European paper products were made from pine. Bowers et al. (1966) isolated and identified this “paper factor” from balsam fir as the methyl ester of todomatuic acid (juvabione) (Figure 5). Juvabione proved effective as a JH mimic only for the family Pyrrhocoridae, with other insects, even those in a closely related family Lygaeidae such as the milkweed bug *Oncopeltus fasciatus*, being unaffected. Such specificity gave rise to the hope that selective JH analogs might be found for prevention of metamorphosis of various pestiferous insects that would not harm beneficial insects such as honeybees.

Zoëcon founded in 1968 by Carl Djerassi, an organic chemist at Stanford University, was the first company formed to search for chemicals that had JH action on particular insects which they named insect growth regulators (IGRs). Methoprene (Figure 5) was first registered in 1975 by the Environmental Protection Agency as Altosid and used primarily in mosquito breeding areas as a larvicide. In treated water, larvae did not metamorphose to the adult, thus reducing the population (Staal, 1975). Today methoprene or another more potent IGR, pyriproxyfen (Figure 5), is still used in integrated pest management schemes to control mosquitoes, particularly those that are resistant to chemical insecticides (Walton and Eldridge, 2020). Methoprene is also used in combination with pyriproxyfen (Rust et al., 2016) or adulticides (Rust and Hemsarth, 2019) in flea collars and other flea products to prevent metamorphosis of flea larvae and kill the adults. Unfortunately, resistance to the IGRs has arisen (Parthasarathy et al., 2012), but combinations of IGRs or with other insecticides such as discussed above in flea control have reduced its impact.

## The Tobacco Hornworm (*Manduca sexta*) and Larval Endocrine Physiology

In the 1970s, the tobacco hornworm (*M. sexta*) became popular for the study of insect growth, molting and metamorphosis. *Manduca* thrived on a completely defined diet (Bell and Joachim, 1976) under laboratory conditions and was utilized initially by the laboratories of Carroll Williams, Fotis Kafatos, and Lynn Riddiford at Harvard University and Larry Gilbert’s laboratory first at Northwestern University, then at the University of North Carolina. Jim Truman as a Junior Fellow at Harvard in the Riddiford and Williams groups showed using simple ligation experiments that the release of PTTH from the larval brain occurred during a certain time of day when the larvae were reared under a light:dark cycle (Truman, 1972; Truman and Riddiford, 1974). PTTH then activated the prothoracic glands to release ecdysone that with its biologically active metabolite, 20-hydroxyecdysone (20E) (see Riddiford et al., 2001), initiated and orchestrated the subsequent molt. The ligature experiments also appeared to indicate that JH necessary for larval molting was released slightly later than PTTH (Truman, 1972) and that JH was again necessary at the time of head capsule slippage during the molt for normal epidermal and cuticular pigmentation (Truman et al., 1973). Later Fain and Riddiford (1976) showed that the apparent delay of JH for the larval molt was due to the slow activation of the prothoracic glands by PTTH. The chance appearance of a *black* mutant larva in the Harvard tobacco hornworm colony (Safranek and Riddiford, 1975) allowed the development of a sensitive bioassay for JH (Fain and Riddiford, 1975). This assay showed that JH was present in the fourth (penultimate) instar larval hemolymph in declining amounts through the feeding and the molting periods and rose again at ecdysis to the fifth and final larval instar.

Fred Nijhout, a graduate student of Williams, subsequently showed that the final larval instar was dependent on a threshold size attained at the time of ecdysis (Nijhout and Williams, 1974a; Nijhout, 1975a). When the larva surpassed the threshold size for metamorphosis, it fed and grew in the final instar to a critical weight that started the endocrine events leading to metamorphosis (Nijhout and Williams, 1974b; Nijhout, 1975b). Subsequent studies by many on both *Manduca* and *Bombyx* have shown that these events are the decline of JH release by the corpora allata, the rise of a specific esterase to degrade JH in the hemolymph and tissues followed by the release of PTTH once the JH titer was sufficiently low (reviewed in Goodman and Granger, 2005; Hiruma and Kaneko, 2013; Nijhout et al., 2014). The subsequent release of ecdysone from the prothoracic glands was relatively small, but in the absence of JH caused the cessation of feeding and the onset of wandering behavior to search for a pupation site (Dominick and Truman, 1985).

During this period, Gibbs and Riddiford (1977) devised a sensitive *Manduca* larval assay for PTTH and showed that the region of the brain containing the lateral neurosecretory cells had the highest PTTH activity. Subsequently, Larry Gilbert’s laboratory identified the pair of PTTH cells in that region (Agui et al., 1979) and studied the control of the prothoracic glands by PTTH (see reviews by Gilbert et al., 2002; Smith and Rybczynski, 2012). Much later *Manduca* PTTH was cloned and recombinant PTTH shown to be active (Gilbert et al., 2000; Shionoya et al., 2003), and *in situ* hybridization of its mRNA showed that only this pair of cells contained PTTH mRNA (Shionoya et al., 2003).

In addition, Gilbert’s laboratory and Bhaskaran’s laboratory at Texas A&M University studied the control of the corpora allata by the brain (Bhaskaran et al., 1980, 1990; see also review by Goodman and Granger, 2005). Both allatotropic and allatoinhibin activities were uncovered, but the allatotropin isolated and sequenced from adult *Manduca* brains was inactive in larvae (Kataoka et al., 1989).

## Cellular Actions of Juvenile Hormone

### Early Studies

The cellular actions of JH were first addressed by Wigglesworth (1940; 1963; 1973) in his studies of its action on the abdominal epidermis of *Rhodnius.* He found that cells responded to the molting hormone at metamorphosis in a particular pattern within the segment and that could be blocked by JH given at different times. He concluded that whereas the molting hormone activates the epidermal cells to begin growth, JH “merely ensures [perhaps by some action at the level of genes (Wigglesworth, 1954), perhaps indirectly by some action upon the cytoplasm (Wigglesworth, 1953)], that the larval pattern is maintained among the activated epidermal cells” (Wigglesworth, 1963). Later Lawrence (1969) found that treatment of *Oncopeltus* with JH at a specific time in the final nymphal instar caused the formation of adult cuticle with larval pigmentation. Willis et al. (1982) extended this study to *Pyrrhocoris*, the Colorado potato beetle, *Leptinotarsa decemlineata*, and various Lepidoptera, showing that there were two effects of JH—one causing a mosaic cuticle which has discrete patches of different stage-specific cuticle due to differing epidermal sensitivity to JH and the other a composite cuticle produced by a single cell which combines features of two metamorphic stages due to differing temporal JH sensitivity of different morphological characteristics.

### Control of Cellular Commitment

The *Manduca* larva provided an epidermis that could be readily cultured and produce cuticle *in vitro* in response to the proper hormonal regimen. A new larval cuticle was synthesized by fourth instar larval epidermis when exposed to 20E immediately after explantation from an intermolt feeding larva (Riddiford et al., 1979, 1980). If, however, cultured in hormone-free media for 24 h, then exposed to 20E, it formed a pupal cuticle (Mitsui and Riddiford, 1978; Riddiford et al., 1980). When final larval instar epidermis was exposed to a low concentration of 20E followed by a high concentration, thereby mimicking the prewandering and pupal molt concentrations of ecdysteroid (Bollenbacher et al., 1981), it formed a pupal cuticle (Mitsui and Riddiford, 1976, 1978). Therefore, one could ask about the direct action of JH on the epidermis under defined conditions.

The abdominal epidermis of *Manduca* is polymorphic in that it first makes a larval cuticle under the influence of JH when exposed to ecdysteroid for the penultimate and final larval molts. Then when exposed to low ecdysteroid in the absence of detectable JH during the pre-wandering peak of ecdysteroid, the epidermis becomes unable to produce a larval cuticle either *in vivo* (when implanted into a penultimate stage larva and allowed to go through final larval molt) (Riddiford, 1976, 1978) or *in vitro* in response to 20E and JH (Mitsui and Riddiford, 1978). Instead it produces pupal cuticle. Hence, in response to the hormonal conditions alone, a single epidermal cell can switch from producing a larval cuticle to a pupal cuticle and therefore is now pupally committed. Although the epidermal cells of beetle (*T. molitor*) (and lepidopteran) larvae are electrically coupled through gap junctions through which small molecules move readily (Caveney and Podgorski, 1975), the cells of the abdominal segment of *Manduca* respond to this ecdysteroid-induced change of commitment in a particular pattern (Truman et al., 1974; Riddiford, 1978) that turns out to be same as the cell-by-cell induction of the Bric-à-brac-Tramtrack-Broad (BTB) transcription factor Broad by 20E (Zhou and Riddiford, 2001).

In *Drosophila* during the molt, 20E initiates a transcription factor cascade called the “Ashburner cascade.” This cascade was first defined by Ashburner et al. (1974) as a series of salivary gland chromosomal puffs (expansions of the polytene DNA strands) induced by 20E *in vitro* that corresponded to the puffs seen *in vivo* at the time of wandering just before and during pupariation. Many of the puffs were later found to encode transcription factors (Thummel, 1990). The same cascade of transcription factors is seen in *Manduca* abdominal epidermis at both the fourth-fifth larval molt and metamorphosis (summarized in Hiruma and Riddiford, 2010) with the exception that Broad is not induced at the fifth larval molt, but only at the time of the ecdysteroid peak that initiates wandering. Thus, *Manduca* larval abdominal epidermis proved to be an ideal system in which to study the action of JH at the cellular and molecular levels. As seen above, when exposed to 20E in the absence of JH, this epidermis first expresses *broad* as it becomes pupally committed. Once committed, it henceforth only makes pupal cuticle when next confronted with a molting concentration of ecdysteroid, whether *in vivo* or *in vitro* (Hiruma and Riddiford, 2010). Once *broad* is activated by the ecdysteroid, its mRNA levels fluctuate during the wandering and prepupal periods directed by the levels of 20E present. Then after pupal ecdysis, *broad* mRNAs disappear from the epidermis over the first 3 days of pupal life and never reappear during the pupal-adult molt or in the adult epidermis (Zhou and Riddiford, 2002). However, when JH is given to the freshly ecdysed pupa, the *broad* transcripts reappear when the ecdysteroid titer arises for the subsequent molt and a “second pupa” rather than an adult is formed. Thus, JH prevents the ecdysteroid-induced switching on of *broad* in the larval epidermis and the ecdysteroid-induced switching off of *broad* in the pupal epidermis.

### *Drosophila* Development, JH and Stage-Specifying Transcription Factors

To elucidate how JH acted to direct the action of ecdysteroid in the polymorphic epidermis, one had to turn to *Drosophila* with its wealth of genetic and molecular biological approaches. *Drosophila* along with the other higher Diptera has however the disadvantage for studying JH action on metamorphosis in that nearly all of the larval tissues except for the Malpighian tubules and the nervous system are discarded at metamorphosis. The adult head and thorax are made from imaginal discs that make larval cuticle and proliferate throughout larval life, then differentiate at metamorphosis, whereas the abdomen comes from abdominal histoblasts (Perez, 1910; Svácha, 1992; Fristrom and Fristrom, 1993). These histoblasts do not divide but make larval cuticle that lies above them during larval life. Then during the prepupal period after pupariation, they begin to divide. The pupal cuticle of the abdomen then is made by reprogrammed larval epidermal cells and histoblasts. After head eversion to form the intact pupa inside the puparium, the proliferated histoblasts spread out from their nests in each abdominal segment, displacing the larval epidermal cells to form the adult epidermis (Ninov et al., 2007, 2009). The displaced larval cells are then engulfed by phagocytic hemocytes (Williams and Truman, 2005). When the ecdysteroid titer rises for the adult molt beginning about 18 h after pupariation (Handler, 1982), the imaginal epidermal cells first respond by undergoing adult commitment and patterning, then as the ecdysteroid titer declines form the adult cuticle beginning about 48–52 h after pupariation (Fristrom and Fristrom, 1993; Zhou and Riddiford, 2002; Ninov et al., 2009).

Normally Broad is present in the histoblasts and their derivatives until about 30 h after pupariation, then disappears (Zhou and Riddiford, 2002). By contrast, when JH is applied at the time of pupariation, the pupa appears normal but the resultant adult is a mosaic of a normal head and thorax and a pupal abdomen (Ashburner, 1970; Postlethwait, 1974). Under these conditions, Broad persists in the adult abdominal epidermis throughout adult development (Zhou and Riddiford, 2002). Furthermore, when *broad-Z1* was overexpressed in the whole animal using a heat shock GAL4 promoter between 30 and 36 h and again at about 48 h after pupariation, a second pupa was formed, indicating that the structures developing from the imaginal discs as well as from the histoblasts were capable of forming pupal cuticle. Thus, the presence of Broad in the cell allows it to make pupal cuticle in response to ecdysteroid and prevents the production of adult cuticle.

During adult development, ecdysone and 20E rise in the absence of JH, and the adult-specifying transcription factor E93 appears (Ureña et al., 2014, 2016). Uyehara et al. (2017) have recently shown that E93 directly acts on the chromatin structure in the enhancers of specific genes, opening up some adult-specific genes such as *nubbin* (important in wing vein formation) for activation and closing the chromatin on others such as *broad*, thus suppressing its expression. Broad and E93 are pupal- and adult-specifying transcription factors respectively throughout the Holometabola (Truman and Riddiford, 2019; Bellés, 2020) and thus fulfill the prediction of Williams and Kafatos (1971) that there are three master regulatory genes that successively activate the larval, pupal, and adult gene sets as metamorphosis proceeds. The idea that the unique features of the larva, pupa, and adult were the results of stage-specific gene sets was nullified by the finding that the same cuticle gene was expressed in the epidermis of two different metamorphic stages to produce a particular type of cuticle (Willis, 1986). This finding however does not negate the hypothesis that there are master regulatory genes that dictate the stage. The stage-specifying transcription factors Broad (pupal) and E93 (adult) along with Krüppel homolog 1 (Kr-h1) in the larva (see below) that regulate each stage and each other (Truman and Riddiford, 2019; Bellés, 2020) seem to be the long-sought regulators.

Williams and Kafatos (1971) also postulated that there is a larval master regulatory gene. *Kr-*h1 may be such a gene. Although initially discovered in *Drosophila* as important for metamorphosis (Pecasse et al., 2000), Kr-h1 is present throughout the insects including the primitive firebrat, *Thermobia domestica* (Konopova et al., 2011), and is necessary to prevent precocious metamorphosis (Minakuchi et al., 2009; Lozano and Belles, 2011). Kr-h1 appears in the embryo at the time that JH appears and is present throughout larval life, then disappears during metamorphosis to the pupa (see review by Truman and Riddiford, 2019). The presence of JH ensures that Kr-h1 will appear when 20E rises for the larval molt and may stabilize Kr-h1 during the larval intermolt period. Kr-h1 disappears at the onset of metamorphosis only to reappear during the molt to the pupa when ecdysteroids again are acting in the presence of JH to prevent adult development of imaginal disc derivatives, the extent of which depends on the species studied. See, for example, the pupal-adult intermediate formed after allatectomy of the Cecropia prepupa (Williams, 1961) versus the normal pupa formed by allatectomized *Bombyx* or *Galleria* larvae (Bounhiol, 1938; Piepho, 1942). However, Kr-h1 does not appear to be a nymph- or larval-specifying factor since its suppression by RNAi in the embryo does not prevent the production of a nymph or larva (Smykal et al., 2014). Instead, the role of Kr-h1 is likely its repression of the expression of the adult-specifying E93 as is seen in both the hemimetabolous cockroach, *Blattella germanica* (Belles and Santos, 2014; Ureña et al., 2016) and the holometabolous *Drosophila* and flour beetle, *Tribolium castaneum* (Ureña et al., 2016). This role of Kr-h1 has been emphasized in naming the molecular pathway underlying the “*status quo*” action of JH in metamorphosis the Met-Kr-h1-E93 (MEKRE93) pathway (Belles and Santos, 2014).

## Juvenile Hormone Receptor

In 1967 when JH was identified as a sesquiterpenoid (Röller et al., 1967), hormones were thought to be only steroids, peptides or proteins with intracellular receptors for the steroids and membrane receptors for the peptides and proteins. The sesquiterpenoid nature of JH allows it to enter the cell (Mitsui et al., 1979) but also allows it to interact with the membrane (Davey, 1996, 2000; Wyatt and Davey, 1996; Goodman and Cusson, 2012). The search for these JH receptors turned out to be long and tortuous and is not over yet.

### Intracellular Receptors

#### JP29

The “*status quo*” action of JH during larval life is assumed to be intracellular since it directs nuclear ecdysteroid action during the larval molts and must be absent for ecdysteroids to initiate metamorphosis. A JH binding protein (JHBP) was isolated from *Manduca* larval hemolymph that bound JH and protected it from the hemolymph esterases but not the JH-specific esterase that appeared once the larva attained the critical weight for metamorphosis (reviewed by Goodman and Cusson, 2012). JHBP is also thought to have other functions including presentation of JH to the cell (see review by Goodman and Cusson, 2012). However, studies *in vitro* showed that JH I alone was just as effective as the combination of JH I and JHBP in the culture medium in preventing the 20E-induced pupal commitment of the *Manduca* abdominal epidermis (Riddiford, 1978).

The search for the intracellular JH receptor in *Manduca* utilized photoaffinity-labeled JHs and JH analogs (synthetic compounds that could be photo-cross-linked to cellular protein(s) to which they bound to allow the subsequent isolation of the protein) (Prestwich et al., 1994). This search yielded a 29 kDa nuclear protein (JP29) which at first was thought to be the JH receptor (Palli et al., 1994). Later studies by Charles et al. (1996) showed that the apparent tight, specific binding of JH I to JP29 was an artifact of a co-purifying esterase that removed the tritiated ester group of JH I used to monitor that binding. Moreover, JP29 was found to bind to the insecticyanin granules in the epidermis and to be regulated by 20E during a larval molt but not after pupal commitment (Shinoda et al., 1997). The role of JP29 in the epidermis is unknown.

#### USP

Ultraspiracle (USP) was shown to be the heterodimeric partner of the ecdysone receptor (EcR) necessary for the action of 20E in tissues, but 20E only bound specifically and tightly to EcR (see review by Riddiford et al., 2001). The *usp* null mutant in *Drosophila* initiates the molt normally at the end of the first instar and makes the second instar cuticle, but is unable to ecdyse (Oro et al., 1992). Rescue to the mid-third instar was afforded by one heat shock pulse of *usp* in the first instar (apparently due to the persistence of USP protein in the larva through the onset of the second instar molt) (Hall and Thummel, 1998). Thereafter, however, the larva entered a “stationary phase” instead of wandering at the expected time of the onset of metamorphosis. Twenty-four hours later, a new cuticle composed of both larval and pupal cuticle proteins covered the posterior region of the body, but the anterior region had only partially everted imaginal discs, and death ensued. Interestingly, the onset of glue protein synthesis in the salivary glands necessary for gluing the puparium to the substrate at the time of pupariation occurred normally at the mid-third instar transition but the glue was not secreted nor did the glands later die as normally occurs. Thus, it appears that USP is needed as a partner for EcR during the larval molts when the ecdysteroid titer is high but is not essential for low ecdysteroid action that causes glue protein synthesis. This latter action may be due to the ecdysteroid sitting on the EcR and causing derepression (Schubiger et al., 2005).

In 1997, Jones and Sharp (1997) found that recombinant *Drosophila* USP specifically bound JH III and JH III acid [the natural JH and its acid metabolite found in *Drosophila* third (final) larval instar hemolymph (Jones et al., 2013)] and suggested that it might be the JH receptor in the larva (Jones and Sharp, 1997; Jones et al., 2006). Unfortunately, the binding of JH III to USP showed about 100-fold lower affinity than one would expect for a hormone receptor. Later studies showed that the potential binding pocket of USP was filled by an unknown phospholipid under *in vivo* conditions (Billas et al., 2001; Sasorith et al., 2002). Also, a *Drosophila* USP-GFP reporter construct that was properly activated by 20E in explanted larval brains and salivary glands was not responsive to JH III alone nor to a combination of 20E and JH III (Beck et al., 2009). Only when the explants were pretreated with JH III before adding the 20E did JH III have an effect—that of reducing the activation produced by 20E. These and other studies (reviewed in Riddiford, 2008) strongly suggested that USP was not the JH receptor during larval life.

Instead USP may be the receptor for methyl farnesoate during the mid-third instar transition in *Drosophila* when methyl farnesoate is the predominant juvenoid in the hemolymph (Jones et al., 2013) and can be converted to JH bis-epoxide (Wen et al., 2015; see Figure 5 for structures of methyl farnesoate and JH bis-epoxide). The Jones group found that USP bound methyl farnesoate with an affinity similar to that of the binding of the retinoid X receptor (RXR) to its ligand 9-*cis*-retinoic acid and that point mutations of USP in residues in the apparent ligand binding pocket decreased this binding 4–10-fold. The *usp*^2^ null mutant could be rescued by USP through metamorphosis but not by the mutant USPs, which instead showed abnormal development at the time of wandering. Thus, the mutated USP apparently could not bind the circulating methyl farnesoate to allow normal metamorphic development although it did not interfere with the normal first and second instar molts. What exact role the methyl farnesoate-USP complex is playing at the onset of metamorphosis remains a mystery that needs exploring.

#### Methoprene-Tolerant (Met) Encodes the JH Receptor

**FIGURE 6:**
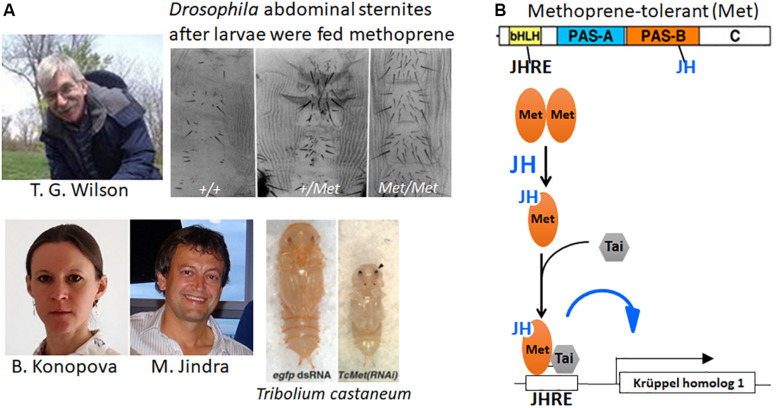
**(A)** *Top:* (Left) Tom Wilson (photo was taken by Emily Wilson and used with her permission). The adult abdominal sternites of the *Methoprene-tolerant* (*Met*^+^) mutant of *Drosophila melanogaster* after the larvae fed on diet containing methoprene (modified from Figure 2 of Wilson and Fabian, 1986). +/+, homozygous wild type; +/*Met*, heterozygous *Met* mutant; *Met*/*Met*, homozygous Met mutant. *Bottom*: Barbora Konopova and Marek Jindra and the precocious pupa they obtained after giving *Met* RNAi to a 4th instar larva of *Tribolium castaneum* (right) compared with a normal pupa that formed after *egfp* RNAi had been given to a 4th instar larva (left) (modified from Figure 3 in Konopova and Jindra, 2007). Photos of Barbora Konopova from her Research Gate file (used with her permission) and of Marek Jindra taken by Masako Asahina (used with Dr. Jindra’s permission). **(B)** *Top*: Cartoon of the Met protein showing its various domains: bHLH, basic helix-loop-helix domain; the Period-Arylhydrocarbon receptor nucleotide translocator-Single-minded (PAS) A and B domains; the C terminal domain. The bHLH domain binds to the juvenile hormone (JH) response element (JHRE) on the target gene promoter; JH binds to the Pas B domains of Met (Charles et al., 2011) and Germ cell-expressed (Gce) [the paralog of Met (Baumann et al., 2010)] (Bittova et al., 2019). *Bottom*: Cartoon of events occurring when JH enters a target cell. The unliganded Met is thought to exist as a homodimer (Godlewski et al., 2006). When JH is present, it binds to Met causing the dissociation of the dimer and the binding of Taiman (Tai) to Met, then both Tai and Met bind to the JHRE on the promoters of Krüppel homolog 1 (Kr-h1) and other JH target genes (Jindra et al., 2015a).

In 1986, Tom Wilson took a different approach to look for the JH receptor. He mutagenized *Drosophila* with ethyl methanesulfonate, then reared the larvae on diet containing a high concentration of the JH analog methoprene (Wilson and Fabian, 1986; Figure 6A, top). Screening with methoprene or JH III that prevents metamorphosis of the abdomen (see above section for JH effects on *Drosophila*) yielded a mutant that was about 100-fold more resistant to JH than the parental wild-type, which they named *Methoprene-tolerant* (*Met*). Because increased levels of JH were needed in the *Met* mutant for both its metamorphic and reproductive effects, they suggested that the Met protein might be involved in JH reception.

This hypothesis was not confirmed until Konopova and Jindra (2007) showed that Met expression was necessary in the flour beetle, *T. castaneum*, to prevent premature metamorphosis. Suppression of Met expression by RNAi in third or fourth instar larvae yielded premature pupation after the fifth or sixth instar (of a total of 7 larval instars) (Figure 6A, bottom). A few of these survived to form miniature adults. Moreover, neither methoprene nor JH III application was effective to cause the formation of “second pupae” by *Tribolium* pupae that had been given Met RNAi. Thus, the loss of Met in *Tribolium* prevented the usual metamorphic responses to JH and therefore fit the criteria for a JH receptor.

As discussed above, the higher Diptera have an extreme form of metamorphosis whereby the larval body is discarded except for the nervous system and the Malpighian tubules; and the adult arises from imaginal cells and discs that have proliferated during larval life or in the case of the abdominal histoblasts during the prepupal period (Perez, 1910; Fristrom and Fristrom, 1993). In *Drosophila* larvae, JH is present and declines before metamorphosis (Bownes and Rembold, 1987; Sliter et al., 1987) but seems to have no role in larval life. Dietary JH caused only a prolongation of the final larval instar followed by a normal appearing puparium that developed to the pharate adult but did not eclose (Bryant and Sang, 1968; Riddiford and Ashburner, 1991). These pharate adults had abdominal defects similar to those found when JH was applied at pupariation (Ashburner, 1970; Postlethwait, 1974) as well as nervous system defects (Restifo and Wilson, 1998; Riddiford et al., 2010, 2018). In contrast to other holometabolous insects, when *Drosophila* larvae were genetically allatectomized by overexpressing cell death genes in the corpora allata, the larvae formed normal puparia but died at or shortly after head eversion to the pupa (Liu et al., 2009; Riddiford et al., 2010). The discrepancy between the *Met* null mutant surviving to the adult and the death of the allatectomized pupa was found to be due to the existence of a second, closely related gene named *germ cell-expressed* (*gce*) (Baumann et al., 2010). *Met–gce* double mutants die at head eversion (Abdou et al., 2011) just as allatectomized prepupae do. *Met* and *gce* are paralogous genes with *gce* representing the ancestral gene (Baumann et al., 2010).

*Methoprene-tolerant* is a member of the basic-helix-loop-helix (bHLH)-Period (per)-Aryl hydrocarbon receptor nuclear translocator (Arnt)-Single-minded (sim) (PAS) domain family of transcription factors (Figure 6B). Charles et al. (2011) found that recombinant Met bound both methoprene and JH III with high affinity in the PAS-B domain. Subsequent studies have shown that the Met-JH complex heterodimerizes with a cofactor Taiman [also known as steroid response coactivator (SRC) and ßFtz-F1 Interacting Steroid Receptor Coactivator (FISC)] and forms a complex that binds to a JH-response element on the mosquito *early trypsin* gene (Li et al., 2011; Jindra et al., 2015a; Liu et al., 2018) and on the *Kr-h1* gene in *Tribolium* and *Blattella* (Zhang et al., 2011; Lozano et al., 2014). Both Met and Gce bind JH III and methyl farnesoate as well as the JH analogs methoprene and pyriproxyfen, and this binding is necessary for the induction of Kr-h1 in *Drosophila* larvae (Jindra et al., 2015b; Wen et al., 2015; Bittova et al., 2019).

### Membrane Receptor

Based on studies with *Rhodnius* follicular cell epithelium (Davey and Huebner, 1974; Davey, 1996), Davey postulated that JH has a membrane receptor for its role in oocyte maturation–increasing the intercellular spaces so that vitellogenin can enter the oocyte. This receptor appeared to activate the sodium-potassium ATPase (“sodium pump”) in the cells causing them to lose water and consequently shrink to create the intercellular spaces (termed “patency”). Abortive attempts were made to isolate this receptor (Davey, 1996). Recently, Jing et al. (2018) showed in the locust that JH causes the phosphorylation of the follicular cell Na-K-ATPase, thus activating it and causing increased patency.

Juvenile hormone activation of the *early trypsin* gene in the mosquito, *Aedes aegypti*, involves the activation of the phospholipase C pathway and the phosphorylation of both Met and Tai which enhances the binding of Met–Tai intracellular dimer on the promoter of the *early trypsin* gene (Liu et al., 2015; Ojani et al., 2016). How this activation of the membrane phospholipids occurs is still unknown.

## Questions for the Future

### Why Is Not JH Required for the First Two Instars in Insects?

In his early experiments, Wigglesworth (1934) parabiosed a decapitated fed first instar nymph to a fed final instar nymph that had been decapitated after the critical period for the adult molt. The first instar nymph molted to a miniature adult with adult abdominal pigmentation and patterning, precocious genitalia and rudimentary wings. Similarly, Piepho (1938b) showed that an integumental (epidermis and overlying cuticle) implant from a first instar *Galleria* larva in a final instar larva formed both a pupal cuticle and an adult cuticle with scales when the host metamorphosed (Figure 2, bottom right). Thus, the cells of early instar animals are capable of forming adult structures when exposed to the proper hormonal environments. Yet under normal conditions in the presence of JH in the immature stages, they form nymphal (*Rhodnius*) or larval (*Galleria*) structures at the molt.

Recent studies have shown, however, that JH is not necessary for progress through the first and second instars after hatching (Daimon et al., 2012; see also reviews by Jindra, 2019; Truman and Riddiford, 2019; Bellés, 2020). Although the corpora allata begin secreting JH about two-thirds of the way through embryogenesis and continue at least to hatching, neither Met nor Kr-h1 is necessary for development of the first or second instar larvae or nymphs respectively of the silkworm *Bombyx* and the linden bug, *Pyrrhocoris* (Smykal et al., 2014; Daimon et al., 2015). Only in the third instar does one begin to see the effects of the absence of JH in terms of the precocious appearance of adult characters in *Pyrrhocoris* or of *broad* mRNA in *Bombyx.* In the latter without the JH receptor Met, most larvae die in the molt to the third instar with mosaic patches of larval and pupal cuticle (Daimon et al., 2015). To resolve this conundrum of the lack of a requirement for JH for the first two instars, Daimon et al. (2015) have proposed that for metamorphosis to occur requires the appearance of a “competence factor” which is necessary to induce *broad* expression. Once this factor appears, the presence of JH at the time of ecdysteroid rise for the molt is necessary to keep *broad* suppressed.

Besides its “*status quo*” action during ecdysteroid-induced molts, JH also is essential for maintaining proliferative growth and suppressing morphogenesis in imaginal cells and discs during the intermolt growth periods of Lepidoptera (Truman et al., 2006). Morphogenetic growth in preparation for metamorphosis normally begins in the final instar when the JH titer declines after the larva has attained the critical weight for metamorphosis (Zhou and Riddiford, 2001; MacWhinnie et al., 2005; Allee et al., 2006). At this time, *broad* mRNA appears in the imaginal cells followed sometime later by the onset of the morphogenetic proliferation. The onset of *broad* expression only requires sucrose feeding, but proliferation requires protein as well (MacWhinnie et al., 2005; Truman et al., 2006; Suzuki et al., 2013). The switch from proliferative growth in the early instars to morphogenetic growth in the final instar requires only the decline of JH and can occur in the absence of ecdysteroid (Truman et al., 2006). However, it requires the presence of a “metamorphosis initiation factor” which is dependent on nutrient input and may be similar to the “competence factor” seen in third instar *Bombyx* larvae (Daimon et al., 2015; Inui and Daimon, 2017). The nutrient input appears to trigger insulin signaling that is necessary for the wing discs to become competent to metamorphose (Koyama et al., 2008; Suzuki et al., 2013).

The requirement for either a nymph or a larva to undergo two feeding instars before metamorphosis holds throughout the insects (Truman, 2019; Truman and Riddiford, 2019). Once they enter the third feeding instar, JH is then necessary to prevent metamorphosis until a threshold size to form a viable adult is achieved. The identities of the “competence factor” for metamorphosis and the “metamorphosis initiation factor” and the necessary interactions between nutrition and hormones to achieve this threshold size are critical problems yet to be solved.

### Why Do *Drosophila* Imaginal Discs and Histoblasts Differ in Their Response to JH?

In higher Diptera, metamorphosis entails the loss of all larval tissues except for the nervous system and the Malpighian tubules (Perez, 1910; Fristrom and Fristrom, 1993). As discussed above in the section on *Drosophila* development, the adult develops externally from the imaginal discs and the abdominal histoblasts and internally from imaginal cells associated with larval tissues. For the epidermis, only the abdominal histoblasts are sensitive to JH, and that sensitivity occurs during their proliferation during the prepupal period. The histoblasts contain both JH receptors, Met and Gce (Baumann et al., 2017). Normally they begin to express *broad* mRNA during the late third instar and continue to do so during their proliferation and spreading until about 24 h after pupariation (Zhou and Riddiford, 2002). Broad protein then disappears from these cells by 30 h after pupariation. When JH was applied at pupariation, Broad protein remained in the abdominal epidermis at least until 72 h after pupariation and these cells make pupal cuticle rather than adult cuticle (Zhou and Riddiford, 2002). Thus, JH seems to be most effective on adult abdominal differentiation when given during the time of histoblast proliferation, but why this is so remains mysterious.

Juvenile hormone has no effect on the metamorphosis of *Drosophila* imaginal discs, even when fed to larvae throughout larval life (Riddiford and Ashburner, 1991) or when given at the time of pupariation or early during the prepupal period (Ashburner, 1970; Postlethwait, 1974). The discs contain only one of the two JH receptors, Met, albeit at low levels; Gce has not been detected in any (Baumann et al., 2017). They however contain the necessary cofactor Taiman. Yet even when *gce* or *gce* and *Met* were overexpressed in the larval wing disc, JH in the diet did not prevent the subsequent metamorphosis of the disc to a normal adult structure (Baumann et al., 2017). This result suggests that the lack of JH effects on imaginal discs is not due to the lack of JH receptors.

For imaginal disc structures, there are two critical periods of adult development: (1) about 30–36 h after pupariation, when morphogenesis of adult hairs and bristles occurs; and (2) about 48–52 h after pupariation just before adult cuticle is deposited (Fristrom and Fristrom, 1993). Timed overexpression of *broad* during these two periods was sufficient to cause the head and thoracic structures to form a “second pupa” (Zhou and Riddiford, 2002), indicating that these imaginal cells can make pupal cuticle. These experiments also suggested that Broad had to be present for pupal differentiation to occur. But why exogenous JH causes high re-expression of Broad in the developing adult abdominal epidermis and only traces of it in the developing adult head and thorax (Zhou and Riddiford, 2002) remains an enigma.

Importantly, when JH was applied at pupariation, Kr-h1 was expressed in the developing adult legs and eyes unlike its lack of expression at this time in control flies (Minakuchi et al., 2008). Thus, these discs can respond to the JH by maintaining Kr-h1 expression but they still undergo metamorphosis. Since they differentiated into normal adult structures, these discs presumably express E93. This presumed E93 expression is counter to the suppression of E93 by Kr-h1 as is normally seen in other species (Belles and Santos, 2014; Ureña et al., 2016). Further elucidation of the interaction of JH, Kr-h1, Broad, and E93 in different *Drosophila* tissues during development and metamorphosis is clearly needed.

### How Is the Secretion of JH Regulated?

Over the years, we have learned much about how the synthesis and secretion of JH is regulated in both immature insects and adults by hormones (neuropeptides, ecdysteroids, and JH itself) and nutritional signals (see reviews by Stay and Tobe, 2007; Goodman and Cusson, 2012; Hiruma and Kaneko, 2013; Marchal et al., 2013; Noriega, 2014; Santos et al., 2019; Bendena et al., 2020). The mechanisms involved in this regulation such as the role of microRNAs are only beginning to be studied (Bendena et al., 2020). Particularly important is a deeper understanding of this regulation by environmental (both internal and external) signals in the role of JH in the control of polyphenisms ranging from pigmentary changes to organization of insect societies (Nijhout, 1995; Miura, 2019).

## Other Roles of JH

Juvenile hormone has a myriad of actions in insects beyond its well-known effects on insect metamorphosis and reproduction. This diversity of action may stem at least partially from its uniqueness among animal hormones in terms of its chemical structure as a sesquiterpenoid and its receptor as a member of the bHLH family of transcription factors. The immediate precursor of JH, methyl farnesoate, is found in the Crustacea which are ancestral to the insects and is involved in reproductive maturation and possibly in early development (Nagaraju, 2011; Qu et al., 2018; Miyakawa et al., 2018). In the crustacean *Daphnia magna* where methyl farnesoate is necessary for environmental male sex determination (Olmstead and Leblanc, 2002; LeBlanc and Medlock, 2015; Toyota et al., 2015), the Met receptor for methyl farnesoate was found to differ in only one amino acid in its binding pocket from the insect *Tribolium* Met (Miyakawa et al., 2013, 2018). This difference was found to favor the binding affinity for methyl farnesoate over JH III.

## Summary

**FIGURE 7:**
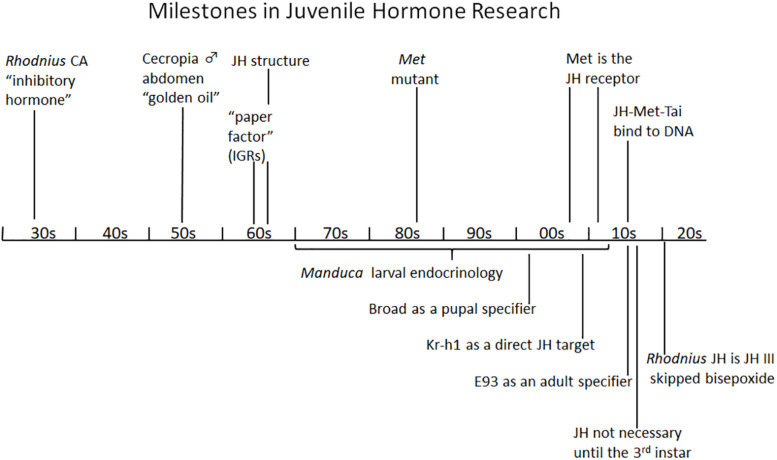
Summary diagram of milestones in juvenile hormone research. See text for details. CA, corpus allatum; IGR, insect growth regulator; JH, juvenile hormone; Kr-h1, Krüppel homolog 1; Met, *Methoprene-tolerant*; Tai, Taiman.

Figure 7 shows the major milestones in the progress of research on JH from the time of its discovery by Wigglesworth in the 1930s to the present which have been detailed in this short review. This progress has been from the phenomenological to the underlying molecular basis of its action. The JH I and II of Cecropia were identified in 1967 and 1968, then the JH III of most other insects as well as JH 0 in Lepidoptera and JH III bis-epoxide in the higher Diptera were identified in the 1970s and 1980s, but it was not until 2020 that the JH of *Rhodnius* was shown to be JHSB3 (Villalobos-Sambucaro et al., 2020). Now that we have the receptors and know something about its downstream effectors, we should be able to delve further into the molecular modes of action of this unique hormone that controls so many aspects of the insect’s life history.

## Author Contributions

LR was the sole author of this manuscript.

## Conflict of Interest

The author declares that the research was conducted in the absence of any commercial or financial relationships that could be construed as a potential conflict of interest.
